# The Tramp Journal

**Published:** 1892-11

**Authors:** 


### THE TRAMP JOURNAL.

It seems that all trades and callings have their tramps. The tramp printer is, the world over, called a "rat," the tramp laborer a "scab"; and this element, pervading every sphere,—commerce, science, art, literature,—is the element of discord and strife. Labor is honorable and dignified; the honest laborer is worthy of his hire. It is the tramp who, by a willingness to work for less wages than an honorable and industrious man with a family and a home to care for can afford to work, creates strikes and labor riots and lock-outs and bloodshed.

And these fellows penetrate everywhere. There are even tramp ships; vessels out of a job, with no regular employment, no regular route. They loaf around and pick up a job (a kind of a cold-victuals job) wherever they can.

But *Journalism* is the latest field to be invaded by this omnipresent *genus;*—yes, we have now the tramp journal, a wolf in sheep's clothing. It has gotten a clean suit of clothes, and although that only covers a price list and an advertisement of a drug manufacturing establishment, it puts on airs and claims to be a "*journal.*" "Burk's Bulletin," it calls itself. Gotten up mechanically in good shape, and containing a sprinkling of literary matter, picked up here and there, it is rather calculated to deceive the unwary. One is apt to overlook the fact that it is only a trade list or catalogue, such as most manufacturers issue and are glad to send free on application, and who pay postage on it as such; one is apt to overlook the fact that the very small amount asked for "subscription" is just that much contributed to the publisher's *advertising fund.* This fact was made perfectly clear, recently, by the announcement that hereafter "*Burk's Bulletin*" would be only one dollar a year, and that the one dollar is not wanted nor expected till the end of the year! A *journal* (?) of eighty or one hundred pages (drug price-list, mostly, it is true) one year for one one dollar, and on a year's credit! It is very clearly to be seen that this "subscription" is a mere shut-eye,—they, the proprietors, would no doubt be glad to send it for nothing to anybody who will take it. (Four "sample copies" of the October number were sent us.) Of course they would; but every gudgeon who pays, or promises to pay, a dollar for it, gets his name on their list, and he is counted as a *subscriber;* hence, all those to whom "sample copies" would be sent any how, are counted as subscribers, and the postal laws are *satisfied* and other advertisers gulled;—the drug price-list goes through the mail as second class matter, at one cent a pound. It is a pretty shrewd trick, successfully worked upon the gullibility of doctors. A "doctor" will pay a dollar for "*Burk's Bulletin,*" and "can't afford" to pay two dollars for a medical journal, even though it be his own State journal. This is bastard competition. Already so many "sample copies" are regularly supplied to the entire list of Texas doctors—and other States too, we presume—that one may very truthfully say, when solicited to take his home journal, "Doctor, I like your journal very much; but I get more medical journals for nothing, than I have time to read." (He gets *Burk's Bulletin* and the *Medical Grief,* no doubt, and they fill the measure of his needs.)

And so it goes. The legitimate journalist who strives to advance the science of medicine; to elevate and promote medical education, and to maintain a high standard of professional character; who keeps his readers informed on all the advances and progress in medicine, and upon all medical topics, and medical or medico-political news of his State, etc., is ready to exclaim with Bill Nye, "we are ruined by (tramp) cheap labor." It is a pretty come to pass when the average medical man is duped into taking a trade list and paying even a dollar for it, and is apparently satisfied with it.

Let me see. Should not the Honorable John Wanamaker's attention be called to this dodge? Did we not see somewhere that a publication issued by the manufacturers or dealers in any of the articles advertised in its pages would not be admitted to the mails at pound rates? Something of the kind occurs to us.

The publishers of legitimate medical journals should do as all other classes do: they should organize a "union" for mutual protection; and all "scabs" or "rats" should be made to occupy the position where they belong,—should set the dogs on them when they come nosing around. Why, there was one publisher who offered to send his paper *free* to all our subscribers, delinquents and all, if we would only advertise the fact! Had that thing the right to the United States mails as second class matter? And so it is; there are those who having no *bona fide* subscription list get names by any kind of dodge—*mere consent* to let the thing be sent *free* will do,—and boast of a big subscription list.

The tramp journal is the curse of our guild, brethren. Let us hear from Wile and Sim and Love and Culbertson on him.

